# Squibb’s Materia Medica

**Published:** 1908-01

**Authors:** 


### Squibb's Materia Medica

FIFTY years ago Dr. E. R. Squibb, at the instigation of Dr. R. S. Satterlee, chief medical purveyor of the United States Army, established in Brooklyn a laboratory to supply the medical department of the army such products as might be required of him. Unfortunately, this laboratory was destroyed by fire in 1858, due to an explosion of ether, which so severely burned Dr. Squibb, himself as to disfigure him for life as well as cause him much suffering as long as he lived. The laboratory, thanks to the financial aid of the medical profession, was rebuilt in 1859, and has continued in operation since that time.

The writer well recalls when, as a young assistant surgeon in the army, he made his first requisition for drugs and appliances in 1861, and which was filled for the most part with Squibb's preparations. Especially is it remembered that during the entire war of 1861-5, Squibb's chloroform and ether were the only anesthetics employed by the writer. And in later years his acquaintance with Dr. Squibb, whom he first met at the meetings of the Medical Society of the State of New York, became one of the pleasantest memories of his life. No one familiar with the genial presence and manner of Dr. Squibb in those days could ever forget him.

Half a century of scientific pharmacy has made the name of Squibb famous throughout the world. Since the death of Dr. Edward R. Squibb the business has been carried on by his sons under the firm name of E. R. Squibb & Sons, and it is pleasant to know that the sterling principles of the father have descended to the sons, and that the firm name stands now for quite as much that is honest, scientific and efficient as ever. American medicine owes a debt of gratitude to the name of Squibb, and in token of fifty years of successful effort this famous house has issued a new edition of Squibb's Materia Medica, including a price list, which is the best pocket edition relating to this topic that has ever been published in English. Whatever bears the name of Squibb in drugs or pharmaceuticals may be relied upon for its intrinsic worth and purity. We beg to offer our congratulations to this distinguished house upon attaining its golden jubilee.

